# Full-Length L1CAM and Not Its Δ2Δ27 Splice Variant Promotes Metastasis through Induction of Gelatinase Expression

**DOI:** 10.1371/journal.pone.0018989

**Published:** 2011-04-25

**Authors:** Stephanie Hauser, Laura Bickel, Dirk Weinspach, Michael Gerg, Michael K. Schäfer, Marco Pfeifer, John Hazin, Florian Schelter, Ulrich H. Weidle, Juliane Ramser, Juliane Volkmann, Alfons Meindl, Manfred Schmitt, Florian Schrötzlmair, Peter Altevogt, Achim Krüger

**Affiliations:** 1 Institut für Experimentelle Onkologie und Therapieforschung Klinkum rechts der Isar der Technischen Universität München, München, Germany; 2 Institut für Anatomie und Zellbiologie, Zentrum für Neurowissenschaften der Albert-Ludwigs-Universität Freiburg, Freiburg, Germany; 3 Abteilung D015 für Translationale Immunologie des Deutschen Krebsforschungszentrums, Heidelberg, Germany; 4 Division Pharma der Roche Diagnostics GmbH, Penzberg, Germany; 5 Frauenklinik und Poliklinik, Klinikum rechts der Isar der Technischen Universität München, München, Germany; Tsan Yuk Hospital, Hospital Authority, China

## Abstract

Tumour-specific splicing is known to contribute to cancer progression. In the case of the L1 cell adhesion molecule (L1CAM), which is expressed in many human tumours and often linked to bad prognosis, alternative splicing results in a full-length form (FL-L1CAM) and a splice variant lacking exons 2 and 27 (SV-L1CAM). It has not been elucidated so far whether SV-L1CAM, classically considered as tumour-associated, or whether FL-L1CAM is the metastasis-promoting isoform. Here, we show that both variants were expressed in human ovarian carcinoma and that exposure of tumour cells to pro-metastatic factors led to an exclusive increase of FL-L1CAM expression. Selective overexpression of one isoform in different tumour cells revealed that only FL-L1CAM promoted experimental lung and/or liver metastasis in mice. In addition, metastasis formation upon up-regulation of FL-L1CAM correlated with increased invasive potential and elevated Matrix metalloproteinase (MMP)-2 and -9 expression and activity *in vitro* as well as enhanced gelatinolytic activity *in vivo*. In conclusion, we identified FL-L1CAM as the metastasis-promoting isoform, thereby exemplifying that high expression of a so-called tumour-associated variant, here SV-L1CAM, is not *per se* equivalent to a decisive role of this isoform in tumour progression.

## Introduction

The L1 cell adhesion molecule (L1CAM), a member of the immunoglobulin-like superfamily of cell adhesion molecules [1], has been shown to be expressed in different isoforms arising through alternative splicing of its mRNA [2]. Alternative splicing of mRNAs is a fine-tuned regulatory mechanism of gene expression during ontogenesis [3]. Over the last years, it has become evident that alternative splicing is frequently deregulated in malignant tumours [4], [5]. Consequently, the expression profile of the various protein isoforms is often different from normal tissues [6] [4]. Importantly, splice variants can exert different [7] [5] or even opposite [8] [6] functions in comparison to their full-length counterparts. Nevertheless, the specific impact of alternative splicing products, including those of L1CAM, on tumour progression has not been fully elucidated so far.

Alternative splicing of the L1CAM mRNA results in a full-length form (FL-L1CAM) and an evolutionary highly conserved splice variant (SV-L1CAM), lacking exons 2 and 27 [9]. The FL-L1CAM variant consists of six immunoglobulin-like domains (Ig1-6), five fibronectin type III repeats, and a short cytoplasmic tail. The SV-L1CAM variant exhibits alterations in the molecular structure N-terminal of the Ig1 region and in the cytoplasmic tail as compared to the full-length L1CAM molecule. In specific, expression of the exon 2 peptide sequence comprising only five amino acids affects homophilic and heterophilic binding to neural ligands [10], [11] which are important for growth-promotion of neural cells [12]. The cytoplasmic sequence encoded by exon 27 is a YRSLE motif which is necessary for clathrin-dependent endocytosis and for regulation of L1CAM density at the cell surface [13]. Indeed, internalization of L1CAM was shown to be important for downstream signaling [14]. Moreover, src-mediated phosphorylation of the tyrosine in the YRSLE motif represents a critical regulatory point of L1CAM-mediated adhesion and intracellular signaling [15].

With regard to tumour pathology, overexpression of L1CAM is detected in a variety of cancers and associated with tumour growth and metastasis [16], [17], [18]. Consequently, elevated levels of L1CAM often indicate bad prognosis for cancer patients [19], [20], [21], [22]. Furthermore, L1CAM has been proposed as a promising therapeutic target since treatment with anti-L1CAM antibodies has been shown to exhibit significant anti-metastatic effects [23], [24], [25]. Importantly, in none of the previous studies about the contribution of L1CAM to tumour progression, the specific roles of FL-L1CAM and SV-L1CAM have been distinguished. This lack of evidence might be due to the general assumption that FL-L1CAM expression was restricted to neuronal tissues [26], whereas SV-L1CAM was detected in non-neuronal tissues including tumours and lymphocytes [27], [28], [29].

In the present study, we revised this axiom by demonstrating that FL-L1CAM and SV-L1CAM mRNAs are both expressed in benign ovarian tumours and both increased during progression of human ovarian carcinomas. Furthermore, incubation of different cancer cells with recombinant Hepatocyte growth factor (recHGF) or Transforming growth factor-β_1_ (recTGF-β_1_), respectively, both known to promote metastasis [30], [31], exclusively increased the expression of FL-L1CAM. We further elucidated that overexpression of FL-L1CAM but not of the splice variant SV-L1CAM conferred increased metastatic potential to tumour cells of three different entities. We showed that elevated expression of FL-L1CAM triggered experimental liver and/or lung metastasis of a human ovarian carcinoma cell line (SKOV3ip-*lacZ*), representing an epithelial solid tumour, of a human fibrosarcoma cell line (HT1080*lacZ*-K15), representing a mesenchymal solid tumour, and of a murine T-lymphoma cell line (L-CI.5s), representing a haematological malignancy. FL-L1CAM-associated tumour progression was accompanied by increased invasive potential and elevated expression and activity of the Matrix metalloproteinases (MMP) -2 and -9, both known to promote metastasis [32]. Taken together, we characterized FL-L1CAM and not the so-called tumour-associated splice variant as the metastasis-promoting isoform of L1CAM.

## Results

### Expression of both, FL-L1CAM and SV-L1CAM, correlated with progression of human ovarian carcinoma

**Figure 1:**
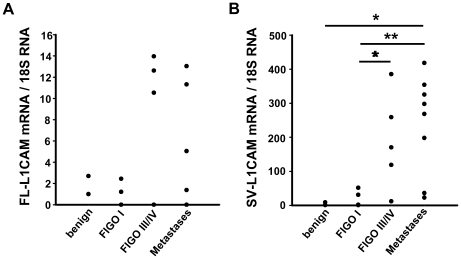
Both FL-L1CAM and SV-L1CAM were expressed in benign and malignant tumours of human ovarian carcinoma patients. Expression of FL-L1CAM (**A**) and SV-L1CAM mRNA (**B**) was analyzed in benign and malignant ovarian tumours and their peritoneal metastases. Mean of relative target gene mRNA *vs.* 18S rRNA ± SEM: **A**. Values obtained for FL-L1CAM; benign tumours: 1.85±0.85, *n* = 2; FIGO I: 0.73±0.49, *n* = 5; FIGO III or FIGO IV: 5.30±2.53, *n* = 7; metastases: 2.57±1.37, *n* = 12 (*p* = 0.664, as determined by Kruskal-Wallis One Way ANOVA on Ranks). **B**. Values obtained for SV-L1CAM; benign tumours: 4.99±3.99, *n* = 2; FIGO I: 17.33±10.41, *n* = 5; FIGO III or FIGO IV: 189.13±63.25, *n* = 5; metastases: 240.21±51.18, *n* = 8 (all groups: *p = 0.015, as determined by Kruskal-Wallis One Way ANOVA on Ranks and subsequent *post hoc* comparison using Holm-Sidak method; pairwise comparisons (unadjusted *p* values): FIGO I vs. benign: *p* = 0.903; FIGO III or FIGO IV vs. benign: *p* = 0.084; metastases vs. benign: *p = 0.024; FIGO III/IV vs. FIGO I: *p = 0.037; metastases vs. FIGO I: **p = 0.005; metastases vs. FIGO III/IV: *p* = 0.465).

To assess whether FL-L1CAM, SV-L1CAM, or both were expressed in tissue samples from cancer patients, we determined their mRNA levels by qRT-PCR in benign and malignant human ovarian tumour samples of different FIGO stages and ovarian cancer peritoneal metastases. The data show that FL-L1CAM and SV-L1CAM mRNAs are both expressed in benign ovarian tumours and both increased during progression of human ovarian carcinomas and were highest in ovarian carcinoma peritoneal metastases (Fig. 1A and B).

### L1CAM splicing was deregulated in favour of FL-L1CAM expression in ovarian and colorectal carcinoma cells upon exposure to pro-metastatic factors

**Figure 2:**
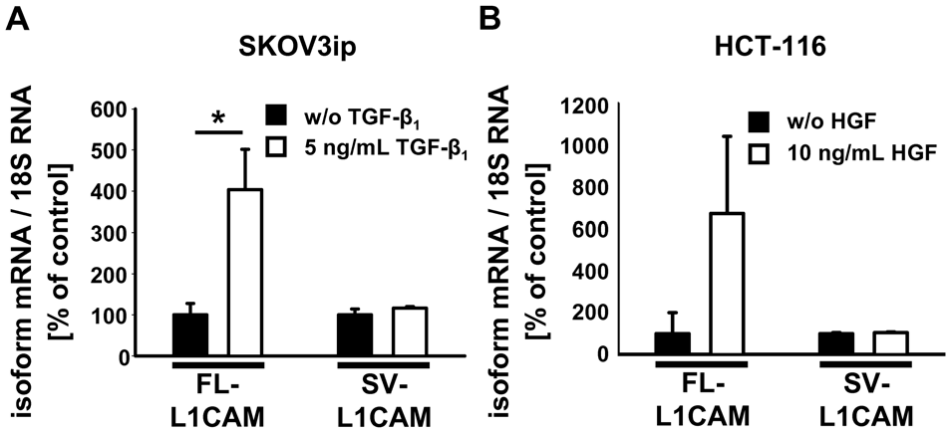
Expression of L1CAM splice variants was deregulated in carcinoma cells upon exposure to pro-metastatic factors. Mean FL-L1CAM or SV-L1CAM mRNA levels ± SEM (*columns* ± *bars*) after incubation with pro-metastatic factors. Target gene mRNA levels were normalized to 18S rRNA levels and the means of the relative reference group without incubation with a pro-metastatic factor were set as 100%. **A**. 1×10^5^ SKOV3ip-*lacZ* ovarian carcinoma cells were incubated for 48 h with or without 5 ng/ml of recombinant TGF-β_1_ (recTGF-β_1_). FL-L1CAM/Ø: 100.0%±27.6%, *n* = 9 cell pools; FL-L1CAM/recTGF-β_1_: 403.5%±98.1%, *n* = 9 cell pools; SV-L1CAM/Ø: 100.0%±14.2%, *n* = 9 cell pools; SV-L1CAM/recTGF-β_1_: 116.2%±4.0%, *n* = 9 cell pools (FL-L1CAM, SKOV3ip-*lacZ*+recTGF-β_1_ vs. SKOV3ip-lacZ−recTGF-β_1_: **p = 0.005; SV-L1CAM, SKOV3ip-*lacZ*+recTGF-β_1_ vs. SKOV3ip-lacZ−recTGF-β_1_: *p* = 0.266, as determined by Wilcoxon Signed Rank test). **B**. 1×10^5^ HCT-116 colon carcinoma cells were incubated for 10 days with or without 10 ng/ml of recombinant HGF (recHGF). FL-L1CAM/Ø: 100.0%±100.0%, *n* = 9 cell pools; FL-L1CAM/recHGF: 673.9%±369.7%, *n* = 9 cell pools; SV-L1CAM/Ø: 100.0%±4.6%, *n* = 9 cell pools; SV-L1CAM/recHGF: 104.9%±2.7%, *n* = 9 cell pools (FL-L1CAM, HCT-116+recHGF vs. HCT-116−recHGF: *p* = 0.313; SV-L1CAM, HCT-116+recHGF vs. HCT-116−recHGF: *p* = 0.250, as determined by Wilcoxon Signed Rank Test).

Next, we wanted to determine whether the relative amount of splicing products of L1CAM could be modified by exposure to known pro-metastatic factors. Therefore, we incubated SKOV3ip-*lacZ* human ovarian carcinoma and HCT-116 human colorectal carcinoma cells with TGF-β_1_ or HGF, respectively. In both cell lines, incubation with the respective pro-metastatic factor led to an increase of FL-L1CAM-mRNA levels, while the expression of SV-L1CAM remained unaltered (Fig. 2A and B), suggesting that the FL-L1CAM variant had an impact on tumour progression.

### FL-L1CAM but not SV-L1CAM promoted metastasis of human fibrosarcoma cells

**Figure 3:**
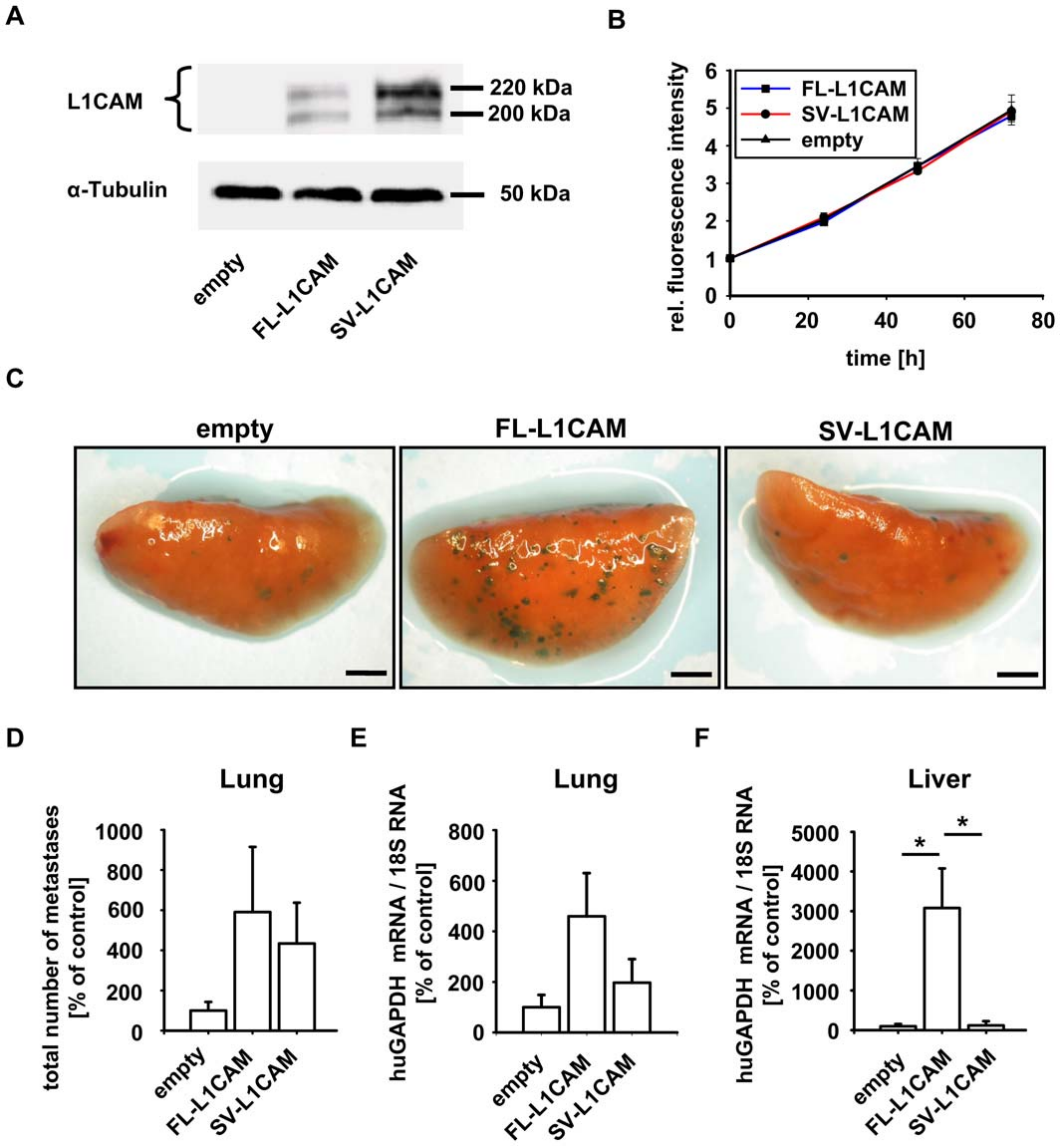
FL-L1CAM promoted the metastatic potential of HT1080*lacZ*-K15 human fibrosarcoma cells. HT1080*lacZ*-K15 cells were transfected with retroviruses coding for FL-L1CAM cDNA or SV-L1CAM cDNA or with an empty vector without transgene. **A**. Western Blot analysis of L1CAM showed that HT1080*lacZ*-K15 cells did not express any L1CAM isoform endogenously, whereas expression of FL-L1CAM or SV-L1CAM was detected after gene transfer. The two bands at 220 kDa and 200 kDa are due to differential glycosylation status of L1CAM. **B**. alamarBlue® proliferation assay did not document any difference in proliferation between the different HT1080*lacZ*-K15 cell lines. Mean cell number ± SEM (*dots* ± *bars*). The mean of the 0 h value within each group was set as 1. Empty: 0 h: 1.000±0.046, 24 h: 2.039±0.037, 48 h: 3.457±0.203, 72 h: 4.950±0.401; FL-L1CAM: 0 h: 1.000±0.014, 24 h: 1.965±0.066, 48 h: 3.458±0.101, 72 h: 4.797±0.164; SV-L1CAM: 0 h: 1.000±0.016, 24 h: 2.096±0.112, 48 h: 3.328±0.031, 72 h: 4.918±0.241. **C** to **E**. 21 days after inoculation of 1×10^6^ of the different HT1080*lacZ*-K15 cells, CD1*^nu/nu^* mice were sacrificed, and their lungs and livers were removed. **C**. X-Gal staining (*indigoblue foci*) of removed lungs. Representative surface images are presented (*bars:* 2 mm). **D**. Mean number of macrometastases in lungs ± SEM (*columns* ± *bars*). The mean of the reference group (*empty*) was set as 100%. Empty: 100.0%±43.3%, *n* = 6 mice; FL-L1CAM: 554.6%±324.1%, *n* = 4 mice; SV-L1CAM: 434.0%±202.8%, *n* = 6 mice (FL-L1CAM vs. empty: *p* = 0.067; SV-L1CAM vs. empty: *p* = 0.180; SV-L1CAM vs. FL-L1CAM: *p* = 0.610, as determined by Mann-Whitney Rank Sum test). **E**. Mean human GAPDH mRNA levels ± SEM (*columns* ± *bars*) in lungs. GAPDH-mRNA levels were normalized to 18S rRNA levels and the mean of the reference group (*empty*) was set as 100%. Empty: 100.0%±48.4%, *n* = 6 mice; FL-L1CAM: 459.1%±171.2%, *n* = 4 mice; SV-L1CAM: 197.1%±92.9%, *n* = 6 mice (FL-L1CAM vs. empty: *p* = 0.067; SV-L1CAM vs. empty: *p* = 0.485; SV-L1CAM vs. FL-L1CAM: *p* = 0.257, as determined by Mann-Whitney Rank Sum test). **F**. Mean human GAPDH mRNA levels ± SEM (*columns* ± *bars*) in livers. GAPDH mRNA levels were normalized to 18S rRNA levels and the mean of the reference group (*empty*) was set as 100%. Empty: 100.0%±56.9%, *n* = 6 mice; FL-L1CAM: 3078.7%±999.7%, *n* = 4 mice; SV-L1CAM: 121.5%±109.2%, *n* = 6 mice (FL-L1CAM vs. empty: *p = 0.010; SV-L1CAM vs. empty: *p* = 1.000; SV-L1CAM vs. FL-L1CAM: *p = 0.019, as determined by Mann-Whitney Rank Sum Test).

In order to investigate a possible metastasis-promoting role of FL-L1CAM, we specifically and stably overexpressed FL-L1CAM or SV-L1CAM in *lacZ*-tagged HT1080 human fibrosarcoma cells (HT1080*lacZ*-K15). As HT1080*lacZ*-K15 cells do not endogeneously express L1CAM (Fig. 3A), we were able to evaluate the impact of FL-L1CAM and SV-L1CAM on metastasis formation without interference through expression of the other isoform. Overexpression was confirmed by Western Blot analysis using an isoform-unspecific antibody (Fig. 3A). Overexpression of neither L1CAM variant influenced tumour cell proliferation (Fig. 3B). Experimental lung metastasis of HT1080*lacZ*-K15 overexpressing FL-L1CAM was increased compared to the control, whereas overexpression of the SV-L1CAM variant was less efficient in inducing lung metastasis (Fig. 3C and D). This visual observation was even more prominent when we performed quantification of metastatic burden by qRT-PCR of human glyceraldehyde-3-phosphate dehydrogenase (GAPDH) as a marker of the human tumor cells (Fig. 3E). In the liver this metastasis-inducing effect of FL-L1CAM overexpression was even markedly superior to SV-L1CAM overexpression, which actually did not promote experimental liver metastasis at all (Fig. 3F).

### FL-L1CAM but not SV-L1CAM also promoted metastasis of human ovarian carcinoma and murine T-lymphoma cells

**Figure 4:**
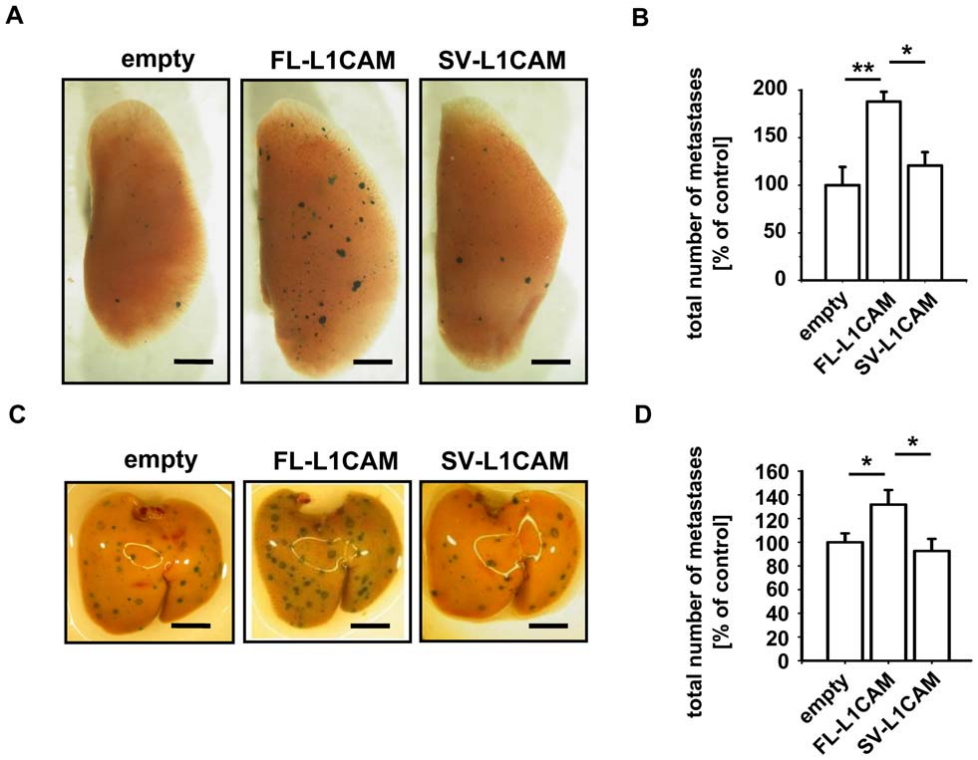
FL-L1CAM also promoted the metastatic potential of SKOV3ip-*lacZ* human ovarian carcinoma and L-CI.5s murine T-lymphoma cells. SKOV3ip-*lacZ* (**A** and **B**) as well as L-CI.5s (**C** and **D**) cells were stably transduced with retroviruses coding for cDNA of FL-L1CAM or SV-L1CAM or with a retroviral vector without cDNA (*empty*). **A** and **B**. 26 days after inoculation of 1.0×10^5^ of the different SKOV3ip-*lacZ* cells, CD1*^nu/nu^* mice were sacrificed and their lungs were removed. **A**. X-Gal staining (*indigoblue foci*) of removed lungs. Representative surface images are presented (*bars:* 2 mm). **B**. Mean number of macrometastases in lungs ± SEM (*columns* ± *bars*). The mean of the reference group (*empty*) was set as 100%; *n* = 4 mice each. Empty: 100.0%±19.1%; FL-L1CAM: 187.8%±10.3%; SV-L1CAM: 120.6%±14.2% (all groups: **p = 0.006, as determined by Kruskal-Wallis One Way ANOVA on Ranks and subsequent *post hoc* comparison using Dunn's method; pairwise comparisons (unadjusted *p* values): FL-L1CAM vs. empty: **p = 0.002; SV-L1CAM vs. empty: *p* = 0.355; SV-L1CAM vs. FL-L1CAM: *p = 0.011). **C**. and **D**. 7 days after inoculation of 5×10^3^ of the different L-CI.5s cells, DBA/2 mice were sacrificed and their livers were removed. **C**. X-Gal staining (*indigoblue foci*) of removed livers. Representative surface images are presented (*bars:* 2 mm). **D**. Mean number of macrometastases in livers ± SEM (*columns* ± *bars*). The mean of the reference group (*empty*) was set as 100%. Empty: 100.0%±7.5%, *n* = 10 mice; FL-L1CAM: 131.8%±12.4%, *n* = 4 mice; SV-L1CAM: 92.6%±10.3%, *n* = 5 mice (FL-L1CAM vs. empty: *p = 0.029; SV-L1CAM vs. empty: *p* = 0.200; SV-L1CAM vs. FL-L1CAM: *p = 0.029, as determined by Mann-Whitney Rank Sum test).

To assess whether the metastasis-promoting effect of FL-L1CAM was cell line-specific, we specifically and stably overexpressed one of the two L1CAM isoforms in *lacZ*-tagged human SKOV3ip-*lacZ* ovarian carcinoma and L-CI.5s T-lymphoma cells. Overexpression of L1CAM variants in both cell lines was confirmed by Western Blot analysis (Figure S1A and S2A). Isoform-transduced SKOV3ip-*lacZ* and L-CI.5s cell lines did not show any difference in cell proliferation as compared to the empty vector controls (Figure S1B and S2B). Lung metastasis of SKOV3ip-*lacZ* (Fig. 4A and B) and liver metastasis of L-CI.5s cells (Fig. 4C and D) were significantly increased only when cells overexpressed the FL-L1CAM variant.

### FL-L1CAM overexpression correlated with increased invasive capacity as well as gelatinase expression and activity

**Figure 5:**
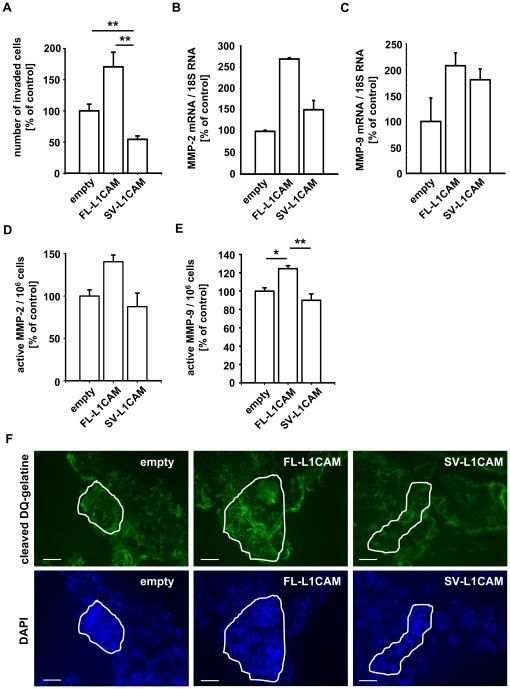
FL-L1CAM over-expression correlated with increased invasive capacity, gelatinase expression and activity *in vitro*, and gelatinolytic activity *in vivo*. HT1080*lacZ*-K15 cells were stably transduced with retroviruses coding for cDNA of FL-L1CAM or SV-L1CAM or with a retroviral vector without cDNA (*empty*). **A**. Mean number of invaded cells per image section ± SEM (*columns ± bars*) for the different HT1080*lacZ*-K15 cells. The mean of the reference group (*empty*) was set as 100%; *n* = 4. Empty: 100.0%±10.6%; FL-L1CAM: 170.6%±23.5%; SV-L1CAM: 54.3%±5.4% (FL-L1CAM vs. empty: *p* = 0.057; SV-L1CAM vs. empty: **p = 0.009; SV-L1CAM vs. FL-L1CAM: **p = 0.003; as determined by Mann-Whitney Rank Sum test). **B** . and **C**. Mean MMP-2 (**B**) and MMP-9 (**C**) mRNA expression levels in the different HT1080*lacZ*-K15 cells ± SEM (*columns* ± *bars*) as quantified by qRT-PCR. The mean of the reference group (*empty*) was set as 100%; *n* = 3. **B**. Empty: 100.0%±2.7%; FL-L1CAM: 269.1%±3.0%; SV-L1CAM: 150.4%±37.6% (FL-L1CAM vs. empty: *p* = 0.100; SV-L1CAM vs. empty: *p* = 0.100; SV-L1CAM vs. FL-L1CAM: *p* = 0.100; as determined by Mann-Whitney Rank Sum Test). **C**. Empty: 100.0%±45.4%; FL-L1CAM: 207.7%±25.0%; SV-L1CAM: 180.7%±20.9% (FL-L1CAM vs. empty: *p* = 0.200; SV-L1CAM vs. empty: *p* = 0.200; SV-L1CAM vs. FL-L1CAM: *p* = 0.400; as determined by Mann-Whitney Rank Sum Test). Mean MMP-2 (**D**) and MMP-9 (**E**) activity of the different HT1080*lacZ*-K15 cells ± SEM (*columns* ± *bars*). The mean of the reference group (*empty*) was set as 100%. **D**. Empty: 100.0%±7.3%; FL-L1CAM: 140.4%±7.9%; SV-L1CAM: 87.5%±15.9%; *n* = 3 (FL-L1CAM vs. empty: *p* = 0.100; SV-L1CAM vs. empty: *p* = 0.700; SV-L1CAM vs. FL-L1CAM: *p* = 0.100; as determined by Mann-Whitney Rank Sum test). **E**. Empty: 100.0%±3.5%; FL-L1CAM: 124.5%±3.3%; SV-L1CAM: 90.0%±7.0%; *n* = 3 (all groups: *p = 0.007, as determined by Kruskal-Wallis One Way ANOVA on Ranks and subsequent *post hoc* comparison using Dunn's method; pairwise comparisons (unadjusted *p* values): FL-L1CAM vs. empty: *p = 0.013; SV-L1CAM vs. empty: *p* = 0.201; SV-L1CAM vs. FL-L1CAM: **p = 0.003). **F**. 21 days after inoculation of 1×10^6^ of the different HT1080*lacZ*-K15 cells, CD1*^nu/nu^* mice were sacrificed and their lungs were removed. *In situ* zymography was performed on cryo-sections of lungs bearing metastases of the different HT1080*lacZ*-K15 cells. Representative images are presented (*bars:* 50 µm; upper row (*green signal*): degraded DQ-gelatine; lower row (*blue signal*): DAPI counter-staining).

In a previous study, L1CAM has been shown to promote invasion of tumour cells [33]. Therefore, we aimed to determine whether overexpression of one of the L1CAM isoforms specifically enhanced the invasive potential of a tumour cell line. We chose the HT1080 model as this cell line does not express endogenous L1CAM, so that the transduced isoforms did not interfere with endogenous L1CAM expression. Overexpression of FL-L1CAM but not of SV-L1CAM led to a significant increase in the invasive potential of this cell line *in vitro* (Fig. 5A). As Matrix metalloproteinases (MMPs) are known to be crucial for the degradation of the matrix in a Matrigel™ invasion assay *in vitro* [34], [35] as well as for the extracellular matrix *in vivo* [36], [37], we wanted to elucidate whether MMPs were involved in FL-L1CAM-induced formation of metastatic colonies, To this end, we determined expression and activity of the gelatinases MMP-2 and MMP-9, well known to be associated with metastasis [38], [39], [40]. Indeed, overexpression of FL-L1CAM in HT1080*lacZ*-K15 cells led to increased expression of MMP-2 (Fig. 5B) and MMP-9 (Fig. 5C). Activity of MMP-2 was markedly, activity of MMP-9 was even significantly increased in tumour cells overexpressing FL-L1CAM *in vitro* (Fig. 5D and E). *In situ* zymography on lung sections showed enhanced gelatinolytic activity within metastatic foci formed by FL-L1CAM overexpressing tumour cells *in vivo* (Fig. 5F).

### FL-L1CAM, and not SV-L1CAM, was preferentially sorted into retrograde and recycling endosomal compartments

 Previous work has demonstrated that the YRSLE motif in the cytoplasmic part of L1CAM encoded by exon 27 mediates clathrin-dependent endocytosis of L1CAM [13]. More recently, compartment-specific endosomal signalling mechanisms of internalized cell surface receptors have been identified [41], suggesting a role of endosomal signalling in development and progression of cancer [42].

**Figure 6:**
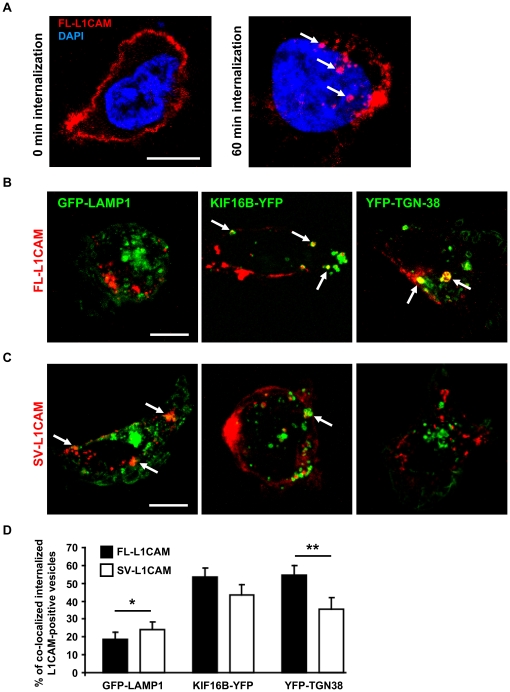
Differential endosomal sorting of FL-L1CAM compared to SV-L1CAM in HT1080*lacZ*-K15 human fibrosarcoma cells. **A**. HT1080*lacZ*-K15 cells overexpressing FL-L1CAM before and after antibody-induced internalization. **B**. and **C**. HT1080*lacZ*-K15 cells 60 min chased at 37°C co-expressing FL-L1CAM or SV-L1CAM together with fluorophore-tagged LAMP1, KIF16B, or TGN38. Arrows indicate internalized L1CAM in selected vesicles that co-localized with compartment markers (Scale bars: 10 µm (**A** to **C**)). **D**. Relative distribution of L1CAM-labeled vesicles in lysosomes (GFP-LAMP1: SV-L1CAM: 24.0%±4.2% SEM; FL-L1CAM: 18.6±4.0% SEM; *p = 0.022, *n* = 30 each), early endosomes (YFP-KIF16B: SV-L1CAM: 43.3%±5.8% SEM; FL-L1CAM: 53.4%±5.1% SEM; *p* = 0.194, *n* = 30 each), retrograde and recycling endosomes (YFP-TGN38: SV-L1CAM: 35.5%±6.3% SEM; FL-L1CAM: 54.7%±5.2% SEM; **p = 0.002, *n* = 20 each) Data was calculated from three (FL- or SV-L1CAM+GFP-LAMP1, *n* = 30 each) or two independent experiments (FL- or SV-L1CAM+YFP-TGN38, *n* = 20 each) and displayed as means ± SEM (*columns* ± *bars*), p values were determined by Mann-Whitney Rank Sum Test.

In order to address this issue, we analyzed in a first attempt the endocytic sorting of FL- and SV-L1CAM following antibody-induced cell surface internalization. We revealed differential endosomal sorting of the L1CAM variants (Fig. 6A to D). We found a significantly higher percentage of vesicles positive for SV-L1CAM that co-localized with GFP-LAMP1-positive lysosomes compared to FL-L1CAM. No significant alterations were observed for FL- or SV-L1CAM positive vesicles that co-localized with YFP-KIF16B-positive early endosomes. However, the ratio of FL-L1CAM-positive vesicles co-localizing with YFP-TGN38 was clearly increased compared to SV-L1CAM. These findings indicate that FL-L1CAM is preferentially sorted into retrograde and recycling endosomes compared to SV-L1CAM. They further suggest that alternative exon usage might result in differential signal propagation from endosomal compartments.

## Discussion

In the present study, we revealed for the first time the different roles of FL-L1CAM and its splice variant in the promotion of metastasis. We demonstrated that the full-length isoform was responsible for the pro-metastatic effect of L1CAM, suggesting an important role of the exons 2 and/or 27 for tumour progression.

L1CAM overexpression is detected in a variety of cancers and is known to promote tumour growth and metastasis [16], [18]. Our present findings that the expression of L1CAM was increased during progression of human ovarian carcinomas are in line with this evidence. Importantly, we detected that also FL-L1CAM is expressed in ovarian tumours of advanced stage, while previous reports emphasized the exclusive tumour-association of SV-L1CAM [29], [43]. Since FL-L1CAM and SV-L1CAM arise from the L1CAM transcript by alternative splicing, one can suggest that factors exist which are able to modulate alternative splicing of L1CAM leading to predominant expression of SV-L1CAM. However, these factors are completely unknown so far. One reason for the neglected significance of FL-L1CAM expression seems to be the fact that it was impossible to distinguish the two isoforms on the protein level due to lack of specific antibodies. Furthermore, possibly due to SV-L1CAM being seemingly tumour-associated, nobody had so far challenged the hypothesis that SV-L1CAM is also functionally the most important L1CAM variant for tumour progression. The surprising finding of the present study is that FL-L1CAM rather than SV-L1CAM is the important determinant of metastasis. This revises the so far prevalent notion that this full length isoform should not play a role in carcinogenesis and tumour progression [28], as – due to its assumed restriction to neuronal tissues [17], [44] – it was thought to be mainly associated with normal physiology or specific neuronal disorders [17]. Importantly, our present finding shows that the FL-L1CAM variant and not the SV-L1CAM splice variant was inducible by exposure of tumour cells to pro-metastatic factors. It has already been reported that the expression of L1CAM can be induced by TGF-β_1_ in general [45], [46]. Interestingly, in the present study we detected a selective up-regulation of FL-L1CAM upon exposure of SKOV3ip-*lacZ* human ovarian carcinoma cells to TGF-β_1_and of HCT-116 human colon carcinoma cells to HGF, which are known pro-metastatic factors [30], [31]. Our study shows that quantity is not the major determinant, as FL-L1CAM, although expressed at lower levels than SV-L1CAM, is still the crucial factor in the specific process of metastatic colonization. Thus, these data allow us to assign a metastasis-promoting function for the previously unsuspected FL-L1CAM rather than for the usual suspect SV-L1CAM. They further support the idea of alternative splicing as a tightly regulated process and suggest that marginal imbalances in the splicing homeostasis, as provoked by pro-metastatic stimuli, are sufficient to elicit or to exacerbate tumours [4], [5], [47].

In order to confirm that FL-L1CAM accounted for the metastasis-promoting function of this adhesion molecule *in vivo*, we inoculated HT1080*lacZ*-K15 that selectively overexpressed either FL-L1CAM or SV-L1CAM. This cell line allows separate functional studies for each isoform without interference through the expression of the other variant, since it does not express any L1CAM variant endogenously. Indeed, elevated levels of FL-L1CAM but not of SV-L1CAM promoted experimental lung metastasis in mice. We ruled out that this metastasis-promoting effect simply relied on increased tumour cell proliferation, a finding which is in accordance with previous results [48], [49]. When we up-regulated FL-L1CAM in human ovarian carcinoma and murine T-lymphoma cells, we observed an analogous promotion of lung (SKOV3ip-*lacZ*) and liver (L-CI.5s) metastasis. This means that the FL-L1CAM-mediated pro-metastatic effect was detectable in different metastatic secondary sites as well as with different tumour entities (solid epithelial, solid mesenchymal, and haematological malignancies), ruling out strict organ- and cell line-specificity. Since the SV-L1CAM isoform was expressed at higher levels than the full length isoform FL-L1CAM upon overexpression in two of the three used cell lines (HT108*lacZ*-K15 and L-CI.5s), one can speculate that the increased metastatic capacity of the FL-L1CAM isoform may be even more pronounced than that detected in the experimental metastasis assay *in vivo*.

As elevated invasive potential is one driving force of metastasis [50] and L1CAM has been shown to promote cell invasion [33], we investigated whether FL-L1CAM caused an increase in the invasive potential of tumour cells. Indeed, we were able to show that overexpression of FL-L1CAM leads to increased invasion of tumour cells *in vitro*. Cell invasion is strongly dependent on proteolytic activity of matrix-degrading enzymes [51]. To reveal a possible link between the FL-L1CAM-specific invasive potential and proteolytic activity, we analyzed whether FL-L1CAM overexpression interfered with the expression and activity of known metastasis-promoting proteases. Indeed, we identified FL-L1CAM as a positive regulator of expression and activity of MMP-2 and MMP-9 *in vitro* as well as of gelatinolytic activity within lung metastases *in vivo*. A link between L1CAM and the induction of proteolytic activity has only been proposed in a wound healing model so far [52]. A possible explanation of the exclusive capacity of the FL-L1CAM to induce MMP gene expression may be found in the altered molecule structure of SV-L1CAM. The lack of exons 2 and 27 leads to a deficient protein sequence in the Ig1 region and the short cytoplasmic tail. This alteration affects not only homophilic and heterophilic adhesions [10] and clathrin-dependent endocytosis [13], it also interferes with signal transduction pathways as L1CAM can induce signalling either through interaction with other molecules [33], [53] or directly *via* its cytoplasmic tail [49], [54], [55].

While we here revealed the expression and increased activity of gelatinases MMP-2 and -9 as one molecular mechanism of how FL-L1CAM executed its pro-metastatic features, the underlying signalling mechanism is still unknown as FL-L1CAM-associated induction of the classical pathways like Erk, NF-κB and STAT3 was not observed (data not shown).

Previous work has demonstrated that alternative use of exon 2 affects homo- as well as heterophilic interactions of L1CAM [11], whereas alternative use of exon 27 affects intracellular sorting upon internalization from the plasma membrane [13]. Both mechanisms might modify signal transduction and subsequent gene expression patterns in cancer cells leading to the observed differences in the metastatic potential and expression of metastasis-associated genes. For example, homophilic interaction of L1CAM activates Erk2 signalling leading to enhanced cell motility and invasion of NIH-3T3 cells [56]. Furthermore, cell-cell adhesion mediated by homophilic L1CAM interaction has been shown to activate other signal transduction pathways including EGF receptor signalling [57]. On the other hand, internalized but not cell surface L1CAM was reported to co-localize with phosphorylated Erk2, suggesting that endocytosis of L1CAM is required for activation of this signalling pathway [14]. Along this line, research on the EGF receptor indicated that signalling mechanisms of the internalized cell surface receptor are distinct from those at the plasma membrane, suggesting that exclusive endosomal localization of certain signalling components is required to facilitate effective signal propagation [58]. Our internalization experiments in HT1080*lacZ*-K15 human fibrosarcoma cells provide evidence for preferential sorting of FL-L1CAM to retrograde endosomal compartments and preferential sorting of SV-L1CAM to lysosomes. Lysosomal sorting of SV-L1CAM may segregate L1CAM from its cytosolic substrates and thereby may be important for negative regulation of pro-proliferative and anti-apoptotic signalling mediated by L1CAM. Conversely, FL-L1CAM may promote survival and proliferation by its preferential endosomal localization. It is therefore possible that alternative splicing of L1CAM activates distinct signalling pathways upon internalization. This issue requires further investigations in future studies.

Taken together, we here show that FL-L1CAM and not the highly expressed SV-L1CAM plays a decisive role in the promotion of metastasis and is responsible for the induction of a metastasis-promoting phenotype in the tumour cell. This finding makes aware that high expression of a so-called tumour-associated variant, here SV-L1CAM, does not *per se* reflect its importance in tumour progression.

## Materials and Methods

### Acquisition of patient samples and ethics statement

Specimens from ovarian tumours and peritoneal metastases of 22 patients were obtained from the Frauenklinik und *Poliklinik*, *Klinikum rechts der Isar der Technischen Universität München*, Germany, and malignant primary tumours were staged according to the present FIGO (*Fédération Internationale de Gynécologie et d'Obstétrique*) classification [59].

The *statutes of the tissue bank* (version from Aug 28, 2007) and the *agreement about asservation of tissue and blood samples for analysis in diagnostics*, *science and education* (version from Jun 19, 2007) regarding the administration of the tissue bank at the *Institut für Allgemeine Pathologie und Pathologische Anatomie*, *Klinikum rechts der Isar der Technischen Universität München*, *Germany*, were approved by the ethics committee of the medical faculty of the *Technische Universität München*. All patients had signed informed consent for the participation to our scientific study.

### Cells and viruses

293T, HCT-116 [60], L-CI.5s [61], L-CI.5s [62], and SKOV3ip-*lacZ* cells [25] were cultured as described previously. Human FL-L1CAM cDNA (NM_000425) was obtained by *Hind*III and *Xho*I digestion of L1a-pcDNA3 (obtained from Vance Lemmon, Laboratory of Axon Growth and Guidance, University of Miami, USA) and subcloned *Hind*III/*Xho*I in the *Hind*III and *Xho*I site of pBluescript-KS(+) (Clontech, Saint-Germain-en-Laye, France). The retroviral plasmid construct with FL-L1CAM cDNA was obtained by *Not*I and *Xho*I digestion and cloned *BamH*I/blunt in the *Not*I and *BamH*I site of pQCXIH/P vectors (Clontech, Saint-Germain-en-Laye, France). Human SV-L1CAM cDNA (NM_001143963) was obtained by *Not*I and *Xba*I digestion of L1Δ(2,27)-pcDNA3 (obtained from Ulrich H. Weidle, *Division Pharma, Roche Diagnostics GmbH*, Penzberg, Germany) and cloned *Pac*I/blunt in the *Not*I and *Pac*I site of pQCXIH/P. Recombinant retroviruses were generated by transient transfection of 293T cells with 10 µg pHIT60 [63], 10 µg pHCMV-G [64] and 10 µg of pQCXIH/P-huFL-L1CAM, or pQCXIH/P-SV-L1CAM, respectively. Retroviral transduction was performed as described previously [61]. Transduction of adherent cells was performed using viral supernatant and transduction of the suspension cell line L-CI.5s was performed by co-culture with virus-producing 293T cells for 48 h.

### Experimental metastasis assays

#### Ethics statement

All animal experiments were performed in compliance with the guidelines of the *Tierschutzgesetz des Freistaates Bayern* and approved by the *Regierung von Oberbayern* (permission number: 55.2-1-54-2531-69-05) and all efforts were made to minimize suffering.

#### Execution

1.0×10^5^ SKOV3ip-*lacZ* or 1×10^6^ HT1080*lacZ*-K15 cells were inoculated into the tail vein of pathogen-free, athymic, female CD1*^nu/nu^* mice (Charles River, Sulzfeld, Germany). 5×10^3^ L-CI.5s were analogously inoculated into pathogen-free, syngeneic, female DBA/2 mice (Charles River, Sulzfeld, Germany). Mice were sacrificed 7 d (L-CI.5s), 21 d (HT1080*lacZ*-K15), or 26 d (SKOV3ip-*lacZ*) after tumour cell inoculation. For quantification of macrometastatic foci, livers or lungs were stained with X-Gal (5-bromo-4-chloro-3-indolyl-β-D-galactopyranoside) (Fermentas, St. Leon-Rot, Germany) as described previously [62]. All foci on the surface of the left lung lobe and foci >0.2 mm on the surface of the median liver lobes were counted.

### *In vitro* assays

For incubation with recHGF or recTGF-β_1_ (both from PromoCell, Heidelberg, Germany), 1×10^5^ cells were seeded on 6 well plates. HCT-116 cells were treated daily with 10 ng/ml recHGF for 10 d. Medium was replaced every second day and confluent cells were trypsinized and transferred to 6 cm dishes. Incubation of SKOV3ip-*lacZ* cells was done for 48 h after addition of 5 ng/ml recTGF-β_1_ on the day after seeding. For analysis of cell viability/proliferation, 2×10^3^ cells/well were seeded in 96 well plates, and the number of living cells was quantified 24 h, 48 h and 72 h after seeding using the alamarBlue® proliferation assay (Invitrogen, Darmstadt, Germany). Analysis was performed according to the manufacturer's protocol. Invasion assays were performed using Costar Transwell Permeable Supports with 8 µm pore size coated with Matrigel™ (Corning Inc., Corning, NY, USA). 5×10^4^ HT1080*lacZ*-K15 cells were seeded in serum-free media. Media containing 10% FCS was used as chemoattractant added to the bottom chamber of 24 well plates. After an incubation time of 24 h at 37°C, non-invasive cells were removed using a cotton stick and invaded cells were fixed using Diff-Quik solution (Dade Behring, Marburg, Germany) and stained using DAPI. For detection of active MMP-2 and MMP-9 in cell culture supernatants, 1.5×10^6^ HT1080*lacZ*-K15 or 1.3×10^6^ SKOV3ip-*lacZ* cells were seeded in a 10 cm dish. After 24 h medium was removed, cells were washed twice with PBS, and incubated with FCS-free medium. Another 48 h later, supernatant was collected and MMP-2 and MMP-9 activities were quantified using either the MMP-2 or MMP-9 Biotrak activity assay system (GE Healthcare, Buckinghamshire, UK) according to the manufacturer's instructions.

### RNA isolation, reverse transcription, and qRT-PCR

According to the manufactures' protocols, RNA isolation from cell lines was performed using TRI-Reagent (Sigma-Aldrich, Taufkirchen, Germany) and RNA isolation from murine lung and liver tissues as well as from human primary tumours and metastases was done with RNeasy Midi Kit (Qiagen, Hilden, Germany) after harvesting the snap-frozen tissues. Reverse transcription and qRT-PCR were performed as described previously [65]. Assays were obtained from Applied Biosystems, Darmstadt, Germany. The inventoried primer-probe-set (Hs01109766_g1) for quantification of FL-L1CAM mRNA transcription detected the exon 26/27 boundary, which is only present in FL-L1CAM (RefSeq: NM_000425.3), whereas the assay ordered on demand for analysis of SV-L1CAM mRNA transcription detected the exon 26/28 boundary (RefSeq: NM_001143963; Assay ID: huL1e26-28-ex26). Detection of absence of exon 27 was sufficient to recognize SV-L1CAM, which is lacking the exons 2 and 27, as splicing of one exon is described to be accompanied by excision of the other exon [66]. For quantification of human MMP-2 and MMP-9 expression, MMP-2 (Hs00234422_m1) and MMP-9 (Hs00234579_m1) TaqMan Gene Expression Assays were used. Human GAPDH was quantified using human GAPD (GAPDH, 4352934E; RefSeq: NM_002046.3). Data was normalized to human 18S rRNA (4319413E, RefSeq: X03205.1).

### Western blot analysis and *in situ* zymography

Protein isolation, purification, and quantification as well as Western Blot analysis were performed as described previously [65]. The monoclonal antibody L1-11A to the ectodomain of human L1CAM (subclone of UJ 127.11) was used for Western Blot analysis of L1CAM as described before [67]. For *in situ* zymography, metastases-bearing lung samples were embedded in Tissue-Tek® O.C.T™ Compound (Sakura Finetek, Staufen, Germany) and shock-frozen on dry-ice. *In situ* zymography was performed as reported previously [68].

### L1CAM internalization assay

HT1080*lacZ*-K15 cells were grown on Poly-D-Lysine (Sigma) coated glass cover slips in DMEM/8% FCS. Cells were transfected by RotifectPlus (Roth, Karlsruhe, Germany) with plasmids encoding either FL-L1CAM or SV-L1CAM along with constructs encoding for GFP-LAMP1 [69], KIF16B-YFP [70] or YFP-TGN38 [71], [72]. Twenty hours after co-transfection, cells were live-stained on ice for 45 min with a polyclonal antibody specific for the extracellular part of human L1CAM [73], which induces L1CAM internalization. Cells were chased for 60 min at 37°C and processed for secondary antibody incubation using Cy3-conjugated anti-rabbit IgG (Jackson ImmunoResearch, West Grove, PA, USA) and confocal microscopy. As a control, we analyzed cells expressing either FL-L1CAM or SV-L1CAM before chase at 37°C, confirming that the plasma membrane of live-stained cells remained intact and that L1CAM-immunolabelling was only observed at the cell surface of transfected cells under these conditions. Double-transfected cells displaying Cy3-labeled L1CAM and low level expression of fluorophore-tagged plasmids were randomly selected and scanned with a LSM 510 confocal microscope (Zeiss, Jena, Germany) using a 63× objective and appropriate filters. Eight confocal middle sections of 0.38 µm were acquired from individual cells. For quantification, *xy*-sections were superimposed as *z*-stacks. The number of the individual intracellular L1CAM-positive vesicles was counted and the percentage of vesicles co-localizing with the fluorophore-tagged marker protein calculated.

### Statistical analysis

Normal distribution of data was tested using the Kolmogorov-Smirnov test and equal variance was tested using Levene-Median test. Three or more group experiments with non-normally distributed data were statistically analyzed by Kruskal-Wallis Analysis of Variance (ANOVA) on Ranks (Fig. 1A) and subsequent *post hoc* comparison by applying either the Holm-Sidak or Dunn's method (all pairwise comparison; Fig. 1B, 4B, and 5E). Two group experiments with non-normally distributed data was analyzed by Mann-Whitney Rank Sum test (Fig. 3E, 3F, 4D, 5A to 5D, and 6D) and Wilcoxon Signed Rank test (in the case of two group comparisons of the same individuals before and after a single treatment; Fig. 2A and 2B). Statistic significance was indicated according to the Michelin Guide scale: *p*<0.05 (*, significant), *p*<0.01 (**, highly significant), and *p*<0.001 (***, extremely significant). All statistical analyses were performed using SigmaStat for Windows version 3.00 software (SPSS Inc.).

## Supporting Information

Figure S1 **L1CAM expression and proliferation analysis of transduced SKOV3ip-** ***lacZ*** **cells.** SKOV3ip-*lacZ* cells were transduced with FL- or SV-L1CAM cDNA or with an empty vector. **A**. Western Blot analysis of L1CAM showed increased expression of L1CAM variants after gene transfer. The two bands at 220 kDa and 200 kDa are due to differential glycosylation status of L1CAM. **B**. Proliferation was unchanged after gene transfer. Data are displayed as mean cell number ± SEM (*dots* ± *bars*). The mean of the 0 h value within each group was set as 1. Empty: 0 h: 1.000±0.014, 24 h: 1.730±0.021, 48 h: 3.090±0.033, 72 h: 4.482±0.188; FL-L1CAM: 0 h: 1.000±0.040, 24 h: 1.604±0.039, 48 h: 2.750±0.070, 72 h: 4.158±0.154; SV-L1CAM: 0 h: 1.000±0.012, 24 h: 1.718±0.034, 48 h: 3.034±0.079, 72 h: 4.598±0.166.
(TIF)

Figure S2 **L1CAM expression and proliferation analysis of transduced L-CI.5s cells.** L-CI.5s cells were transduced with FL- or SV-L1CAM cDNA or with an empty vector. **A**. Western Blot analysis of L1CAM showed increased expression of L1CAM variants after gene transfer. The two bands at 220 kDa and 200 kDa are due to differential glycosylation status of L1CAM. **B**. Proliferation was unchanged after gene transfer. Data are displayed as mean cell number ± SEM (*dots* ± *bars*). The mean of the 0 h value within each group was set as 1. Empty: 0 h: 1.000±0.028, 24 h: 2.761±0.126, 48 h: 7.141±0.838, 72 h: 11.941±0.520; FL-L1CAM: 0 h: 1.000±0.026, 24 h: 2.479±0.166, 48 h: 7.028±0.115, 72 h: 11.071±0.576; SV-L1CAM: 0 h: 1.000±0.065, 24 h: 2.778±0.214, 48 h: 7.406±0.138, 72 h: 12.458plusmn;0.387.
(TIF)

## References

[1] Moos M, Tacke R, Scherer H, Teplow D, Fruh K (1988). Neural adhesion molecule L1 as a member of the immunoglobulin superfamily with binding domains similar to fibronectin.. Nature.

[2] Reid RA, Hemperly JJ (1992). Variants of human L1 cell adhesion molecule arise through alternate splicing of RNA.. J Mol Neurosci.

[3] Breitbart RE, Andreadis A, Nadal-Ginard B (1987). Alternative splicing: a ubiquitous mechanism for the generation of multiple protein isoforms from single genes.. Annu Rev Biochem.

[4] Ghigna C, Valacca C, Biamonti G (2008). Alternative splicing and tumor progression.. Curr Genomics.

[5] Pajares MJ, Ezponda T, Catena R, Calvo A, Pio R (2007). Alternative splicing: an emerging topic in molecular and clinical oncology.. Lancet Oncol.

[6] Skotheim RI, Nees M (2007). Alternative splicing in cancer: noise, functional, or systematic?. Int J Biochem Cell Biol.

[7] Srebrow A, Kornblihtt AR (2006). The connection between splicing and cancer.. J Cell Sci.

[8] Scotlandi K, Zuntini M, Manara MC, Sciandra M, Rocchi A (2007). CD99 isoforms dictate opposite functions in tumour malignancy and metastases by activating or repressing c-Src kinase activity.. Oncogene.

[9] Coutelle O, Nyakatura G, Taudien S, Elgar G, Brenner S (1998). The neural cell adhesion molecule L1: genomic organisation and differential splicing is conserved between man and the pufferfish Fugu.. Gene.

[10] Jacob J, Haspel J, Kane-Goldsmith N, Grumet M (2002). L1 mediated homophilic binding and neurite outgrowth are modulated by alternative splicing of exon 2.. J Neurobiol.

[11] De Angelis E, Brummendorf T, Cheng L, Lemmon V, Kenwrick S (2001). Alternative use of a mini exon of the L1 gene affects L1 binding to neural ligands.. J Biol Chem.

[12] Gouveia RM, Gomes CM, Sousa M, Alves PM, Costa J (2008). Kinetic analysis of L1 homophilic interaction: role of the first four immunoglobulin domains and implications on binding mechanism.. J Biol Chem.

[13] Kamiguchi H, Long KE, Pendergast M, Schaefer AW, Rapoport I (1998). The neural cell adhesion molecule L1 interacts with the AP-2 adaptor and is endocytosed via the clathrin-mediated pathway.. J Neurosci.

[14] Schaefer AW, Kamiguchi H, Wong EV, Beach CM, Landreth G (1999). Activation of the MAPK signal cascade by the neural cell adhesion molecule L1 requires L1 internalization.. J Biol Chem.

[15] Schaefer AW, Kamei Y, Kamiguchi H, Wong EV, Rapoport I (2002). L1 endocytosis is controlled by a phosphorylation-dephosphorylation cycle stimulated by outside-in signaling by L1.. J Cell Biol.

[16] Siesser PF, Maness PF (2009). L1 cell adhesion molecules as regulators of tumor cell invasiveness.. Cell Adh Migr.

[17] Schafer MK, Altevogt P (2010). L1CAM malfunction in the nervous system and human carcinomas.. Cell Mol Life Sci.

[18] Gavert N, Ben-Shmuel A, Raveh S, Ben-Ze'ev A (2008). L1-CAM in cancerous tissues.. Expert Opin Biol Ther.

[19] Schroder C, Schumacher U, Fogel M, Feuerhake F, Muller V (2009). Expression and prognostic value of L1-CAM in breast cancer.. Oncol Rep.

[20] Kaifi JT, Reichelt U, Quaas A, Schurr PG, Wachowiak R (2007). L1 is associated with micrometastatic spread and poor outcome in colorectal cancer.. Mod Pathol.

[21] Fogel M, Gutwein P, Mechtersheimer S, Riedle S, Stoeck A (2003). L1 expression as a predictor of progression and survival in patients with uterine and ovarian carcinomas.. Lancet.

[22] Boo YJ, Park JM, Kim J, Chae YS, Min BW (2007). L1 expression as a marker for poor prognosis, tumor progression, and short survival in patients with colorectal cancer.. Ann Surg Oncol.

[23] Wolterink S, Moldenhauer G, Fogel M, Kiefel H, Pfeifer M (2010). Therapeutic antibodies to human L1CAM: functional characterization and application in a mouse model for ovarian carcinoma.. Cancer Res.

[24] Novak-Hofer I, Cohrs S, Grunberg J, Friedli A, Schlatter MC (2008). Antibodies directed against L1-CAM synergize with Genistein in inhibiting growth and survival pathways in SKOV3ip human ovarian cancer cells.. Cancer Lett.

[25] Arlt MJ, Novak-Hofer I, Gast D, Gschwend V, Moldenhauer G (2006). Efficient inhibition of intra-peritoneal tumor growth and dissemination of human ovarian carcinoma cells in nude mice by anti-L1-cell adhesion molecule monoclonal antibody treatment.. Cancer Res.

[26] Miura M, Asou H, Kobayashi M, Uyemura K (1992). Functional expression of a full-length cDNA coding for rat neural cell adhesion molecule L1 mediates homophilic intercellular adhesion and migration of cerebellar neurons.. J Biol Chem.

[27] Itoh K, Sakurai Y, Asou H, Umeda M (2000). Differential expression of alternatively spliced neural cell adhesion molecule L1 isoforms during oligodendrocyte maturation.. J Neurosci Res.

[28] Takeda Y, Asou H, Murakami Y, Miura M, Kobayashi M (1996). A nonneuronal isoform of cell adhesion molecule L1: tissue-specific expression and functional analysis.. J Neurochem.

[29] Euer NI, Kaul S, Deissler H, Mobus VJ, Zeillinger R (2005). Identification of L1CAM, Jagged2 and Neuromedin U as ovarian cancer-associated antigens.. Oncol Rep.

[30] Lesko E, Majka M (2008). The biological role of HGF-MET axis in tumor growth and development of metastasis.. Front Biosci.

[31] Jakowlew SB (2006). Transforming growth factor-beta in cancer and metastasis.. Cancer Metastasis Rev.

[32] Cairns RA, Khokha R, Hill RP (2003). Molecular mechanisms of tumor invasion and metastasis: an integrated view.. Curr Mol Med.

[33] Gast D, Riedle S, Kiefel H, Muerkoster SS, Schafer H (2008). The RGD integrin binding site in human L1-CAM is important for nuclear signaling.. Exp Cell Res.

[34] Abe T, Mori T, Kohno K, Seiki M, Hayakawa T (1994). Expression of 72 kDa type IV collagenase and invasion activity of human glioma cells.. Clin Exp Metastasis.

[35] Young TN, Rodriguez GC, Rinehart AR, Bast RC, Pizzo SV (1996). Characterization of gelatinases linked to extracellular matrix invasion in ovarian adenocarcinoma: purification of matrix metalloproteinase 2.. Gynecol Oncol.

[36] Davies B, Waxman J, Wasan H, Abel P, Williams G (1993). Levels of matrix metalloproteases in bladder cancer correlate with tumor grade and invasion.. Cancer Res.

[37] Zhang L, Shi J, Feng J, Klocker H, Lee C (2004). Type IV collagenase (matrix metalloproteinase-2 and -9) in prostate cancer.. Prostate Cancer Prostatic Dis.

[38] Togawa D, Koshino T, Saito T, Takagi T, Machida J (1999). Highly activated matrix metalloproteinase-2 secreted from clones of metastatic lung nodules of nude mice injected with human fibrosarcoma HT1080.. Cancer Lett.

[39] Gerg M, Kopitz C, Schaten S, Tschukes A, Kahlert C (2008). Distinct Functionality of Tumor Cell-Derived Gelatinases during Formation of Liver Metastases.. Mol Cancer Res.

[40] Yu Q, Stamenkovic I (2000). Cell surface-localized matrix metalloproteinase-9 proteolytically activates TGF-beta and promotes tumor invasion and angiogenesis.. Genes Dev.

[41] Kim HJ, Taylor LJ, Bar-Sagi D (2007). Spatial regulation of EGFR signaling by Sprouty2.. Curr Biol.

[42] Sebastian S, Settleman J, Reshkin SJ, Azzariti A, Bellizzi A (2006). The complexity of targeting EGFR signalling in cancer: from expression to turnover.. Biochim Biophys Acta.

[43] Shtutman M, Levina E, Ohouo P, Baig M, Roninson IB (2006). Cell adhesion molecule L1 disrupts E-cadherin-containing adherens junctions and increases scattering and motility of MCF7 breast carcinoma cells.. Cancer Res.

[44] Raveh S, Gavert N, Ben-Ze'ev A (2009). L1 cell adhesion molecule (L1CAM) in invasive tumors.. Cancer Lett.

[45] Geismann C, Morscheck M, Koch D, Bergmann F, Ungefroren H (2009). Up-regulation of L1CAM in pancreatic duct cells is transforming growth factor beta1- and slug-dependent: role in malignant transformation of pancreatic cancer.. Cancer Res.

[46] Huszar M, Pfeifer M, Schirmer U, Kiefel H, Konecny GE (2010). Up-regulation of L1CAM is linked to loss of hormone receptors and E-cadherin in aggressive subtypes of endometrial carcinomas.. J Pathol.

[47] Tazi J, Bakkour N, Stamm S (2009). Alternative splicing and disease.. Biochim Biophys Acta.

[48] Gast D, Riedle S, Schabath H, Schlich S, Schneider A (2005). L1 augments cell migration and tumor growth but not beta3 integrin expression in ovarian carcinomas.. Int J Cancer.

[49] Gast D, Riedle S, Issa Y, Pfeifer M, Beckhove P (2008). The cytoplasmic part of L1-CAM controls growth and gene expression in human tumors that is reversed by therapeutic antibodies.. Oncogene.

[50] Geiger TR, Peeper DS (2009). Metastasis mechanisms.. Biochim Biophys Acta.

[51] Bjorklund M, Koivunen E (2005). Gelatinase-mediated migration and invasion of cancer cells.. Biochim Biophys Acta.

[52] Urech L, Bittermann AG, Hubbell JA, Hall H (2005). Mechanical properties, proteolytic degradability and biological modifications affect angiogenic process extension into native and modified fibrin matrices in vitro.. Biomaterials.

[53] Schmid RS, Maness PF (2008). L1 and NCAM adhesion molecules as signaling coreceptors in neuronal migration and process outgrowth.. Curr Opin Neurobiol.

[54] Gavert N, Ben-Shmuel A, Lemmon V, Brabletz T, Ben-Ze'ev A (2010). Nuclear factor-kappaB signaling and ezrin are essential for L1-mediated metastasis of colon cancer cells.. J Cell Sci.

[55] Tyukhtenko S, Deshmukh L, Kumar V, Lary J, Cole J (2008). Characterization of the neuron-specific L1-CAM cytoplasmic tail: naturally disordered in solution it exercises different binding modes for different adaptor proteins.. Biochemistry.

[56] Silletti S, Yebra M, Perez B, Cirulli V, McMahon M (2004). Extracellular signal-regulated kinase (ERK)-dependent gene expression contributes to L1 cell adhesion molecule-dependent motility and invasion.. J Biol Chem.

[57] Islam R, Kristiansen LV, Romani S, Garcia-Alonso L, Hortsch M (2004). Activation of EGF receptor kinase by L1-mediated homophilic cell interactions.. Mol Biol Cell.

[58] Murphy JE, Padilla BE, Hasdemir B, Cottrell GS, Bunnett NW (2009). Endosomes: a legitimate platform for the signaling train.. Proc Natl Acad Sci U S A.

[59] Shepherd JH (1989). Revised FIGO staging for gynaecological cancer.. Br J Obstet Gynaecol.

[60] Brattain MG, Fine WD, Khaled FM, Thompson J, Brattain DE (1981). Heterogeneity of malignant cells from a human colonic carcinoma.. Cancer Res.

[61] Kopitz C, Anton M, Gansbacher B, Krüger A (2005). Reduction of experimental human fibrosarcoma lung metastasis in mice by adenovirus-mediated cystatin C overexpression in the host.. Cancer Res.

[62] Krüger A, Schirrmacher V, von Hoegen P (1994). Scattered micrometastases visualized at the single-cell level: detection and re-isolation of lacZ-labeled metastasized lymphoma cells.. Int J Cancer.

[63] Soneoka Y, Cannon PM, Ramsdale EE, Griffiths JC, Romano G (1995). A transient three-plasmid expression system for the production of high titer retroviral vectors.. Nucleic Acids Res.

[64] Yee JK, Miyanohara A, LaPorte P, Bouic K, Burns JC (1994). A general method for the generation of high-titer, pantropic retroviral vectors: highly efficient infection of primary hepatocytes.. Proc Natl Acad Sci U S A.

[65] Arlt M, Kopitz C, Pennington C, Watson KL, Krell HW (2002). Increase in Gelatinase-specificity of Matrix Metalloproteinase Inhibitors Correlates with Antimetastatic Efficacy in a T-Cell Lymphoma Model.. Cancer Res.

[66] Jouet M, Rosenthal A, Kenwrick S (1995). Exon 2 of the gene for neural cell adhesion molecule L1 is alternatively spliced in B cells.. Brain Res Mol Brain Res.

[67] Mechtersheimer S, Gutwein P, Agmon-Levin N, Stoeck A, Oleszewski M (2001). Ectodomain shedding of L1 adhesion molecule promotes cell migration by autocrine binding to integrins.. J Cell Biol.

[68] Krüger A, Arlt MJ, Gerg M, Kopitz C, Bernardo MM (2005). Antimetastatic activity of a novel mechanism-based gelatinase inhibitor.. Cancer Res.

[69] Falcon-Perez JM, Nazarian R, Sabatti C, Dell'Angelica EC (2005). Distribution and dynamics of Lamp1-containing endocytic organelles in fibroblasts deficient in BLOC-3.. J Cell Sci.

[70] Hoepfner S, Severin F, Cabezas A, Habermann B, Runge A (2005). Modulation of receptor recycling and degradation by the endosomal kinesin KIF16B.. Cell.

[71] Ghosh RN, Mallet WG, Soe TT, McGraw TE, Maxfield FR (1998). An endocytosed TGN38 chimeric protein is delivered to the TGN after trafficking through the endocytic recycling compartment in CHO cells.. J Cell Biol.

[72] Keller P, Toomre D, Diaz E, White J, Simons K (2001). Multicolour imaging of post-Golgi sorting and trafficking in live cells.. Nat Cell Biol.

[73] Schäfer MK, Nam YC, Moumen A, Keglowich L, Bouche E (2010). L1 syndrome mutations impair neuronal L1 function at different levels by divergent mechanisms.. Neurobiol Dis.

