# Supplementary figures and images for: Full-Length L1CAM and Not Its Δ2Δ27 Splice Variant Promotes Metastasis through Induction of Gelatinase Expression
Source: PLoS One. 2011 Apr 25;6(4):e18989. doi: 10.1371/journal.pone.0018989 (PMC3081839; doi:10.1371/journal.pone.0018989)

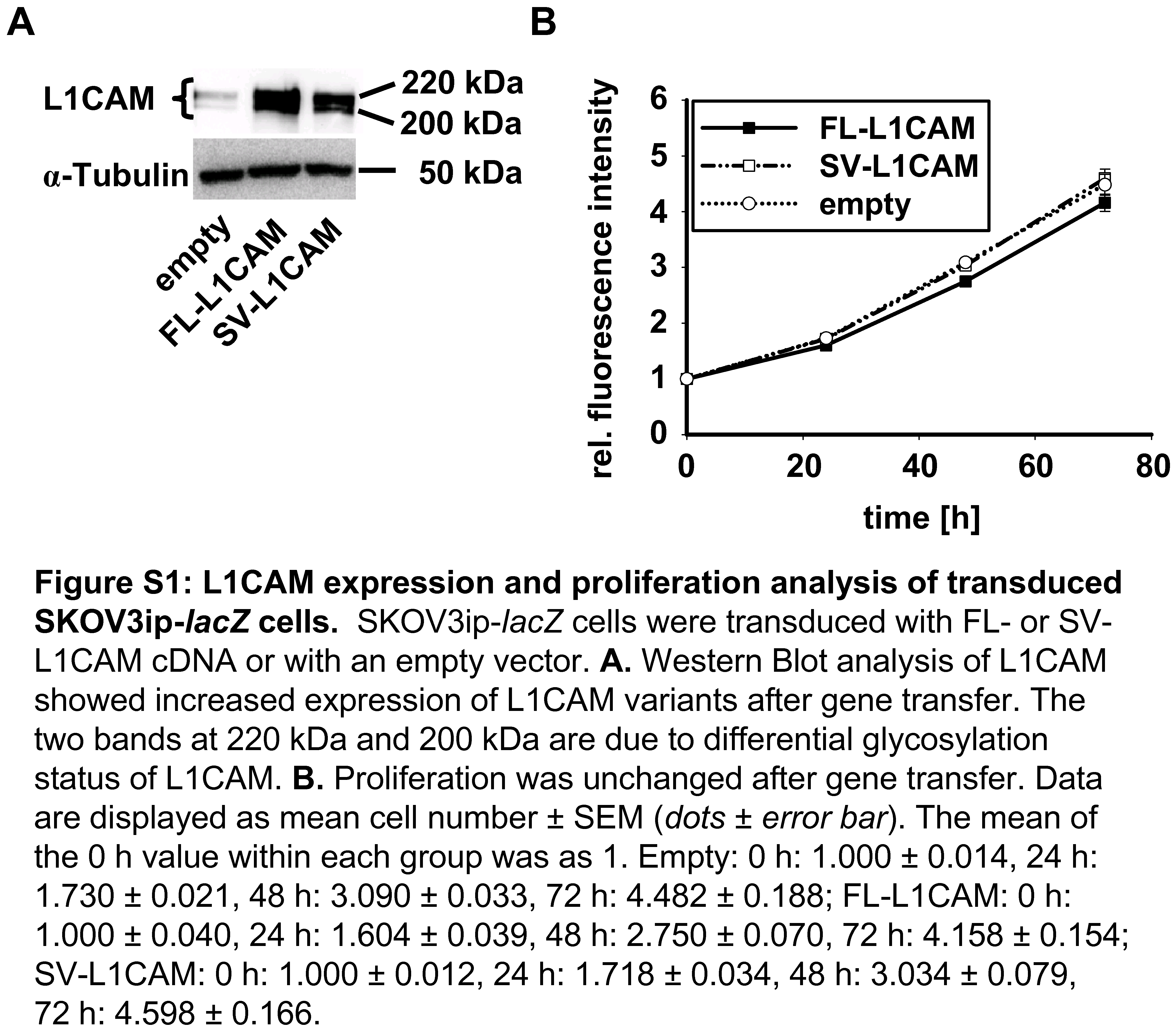

Supplement: Figure S1 — L1CAM expression and proliferation analysis of transduced SKOV3ip- lacZ cells. SKOV3ip-lacZ cells were transduced with FL- or SV-L1CAM cDNA or with an empty vector. A. Western Blot analysis of L1CAM showed increased expression of L1CAM variants after gene transfer. The two bands at 220 kDa and 200 kDa are due to differential glycosylation status of L1CAM. B. Proliferation was unchanged after gene transfer. Data are displayed as mean cell number ± SEM (dots ± bars). The mean of the 0 h value within each group was set as 1. Empty: 0 h: 1.000±0.014, 24 h: 1.730±0.021, 48 h: 3.090±0.033, 72 h: 4.482±0.188; FL-L1CAM: 0 h: 1.000±0.040, 24 h: 1.604±0.039, 48 h: 2.750±0.070, 72 h: 4.158±0.154; SV-L1CAM: 0 h: 1.000±0.012, 24 h: 1.718±0.034, 48 h: 3.034±0.079, 72 h: 4.598±0.166. (TIF) [file pone.0018989.s001.tif]

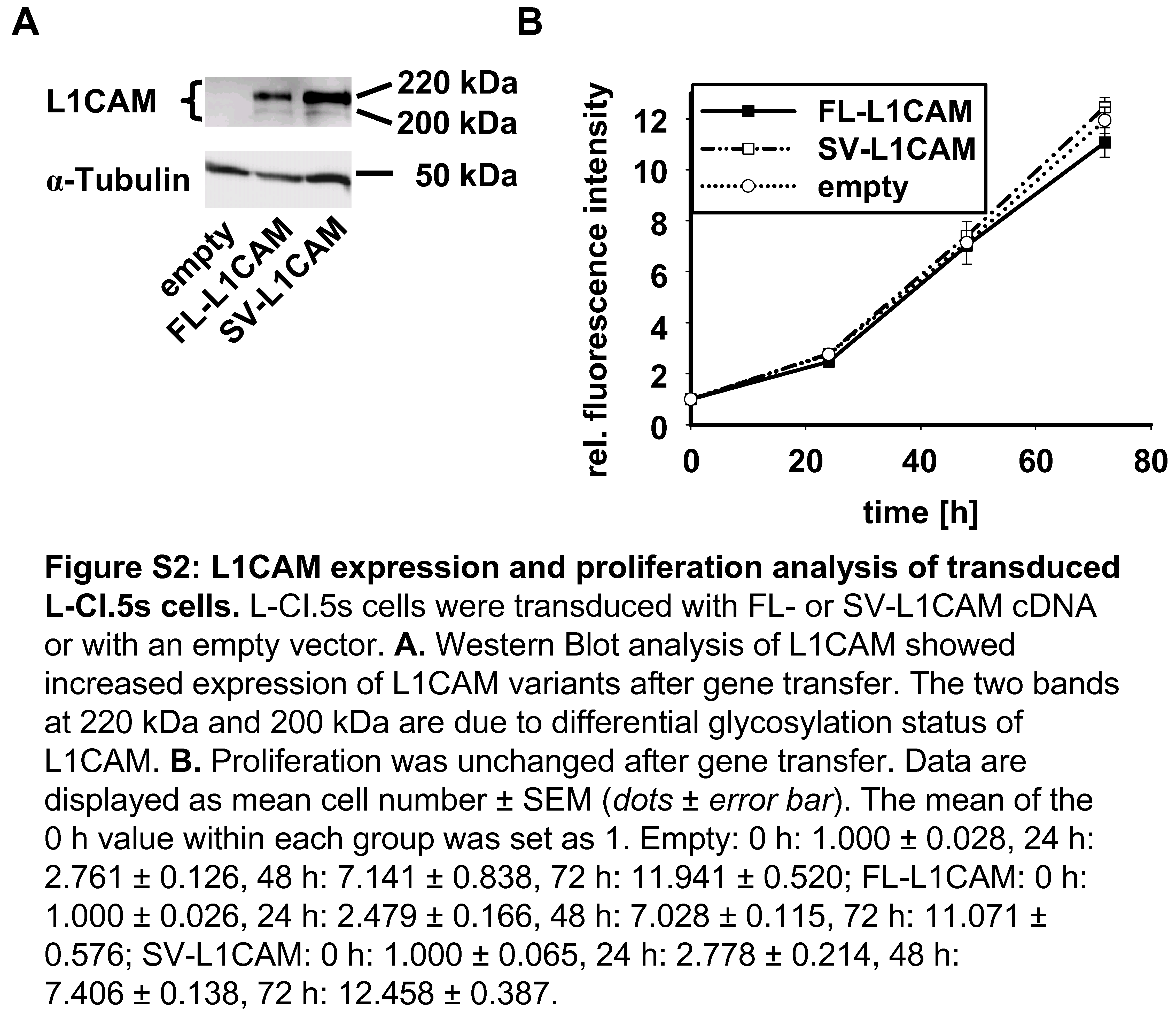

Supplement: Figure S2 — L1CAM expression and proliferation analysis of transduced L-CI.5s cells. L-CI.5s cells were transduced with FL- or SV-L1CAM cDNA or with an empty vector. A. Western Blot analysis of L1CAM showed increased expression of L1CAM variants after gene transfer. The two bands at 220 kDa and 200 kDa are due to differential glycosylation status of L1CAM. B. Proliferation was unchanged after gene transfer. Data are displayed as mean cell number ± SEM (dots ± bars). The mean of the 0 h value within each group was set as 1. Empty: 0 h: 1.000±0.028, 24 h: 2.761±0.126, 48 h: 7.141±0.838, 72 h: 11.941±0.520; FL-L1CAM: 0 h: 1.000±0.026, 24 h: 2.479±0.166, 48 h: 7.028±0.115, 72 h: 11.071±0.576; SV-L1CAM: 0 h: 1.000±0.065, 24 h: 2.778±0.214, 48 h: 7.406±0.138, 72 h: 12.458plusmn;0.387. (TIF) [file pone.0018989.s002.tif]
